# The Impact of Obesity on Durable Left Ventricular Assist Device Implantation: A Systematic Review and Meta‐Analysis

**DOI:** 10.1111/aor.70071

**Published:** 2025-12-12

**Authors:** Hugh Jacobs, Olivia Frost, Arian Arjomandi Rad, Wing Kiu Chou, Sadeq Al‐Saegh, Alina Zubarevich, Alexander Weymann, Arjang Ruhparwar, Lukman Amanov, Peyman Sardari Nia, Antonios Kourliouros, Thanos Athanasiou

**Affiliations:** ^1^ Department of Cardiothoracic Surgery Bristol Heart Institute Bristol UK; ^2^ Department of Cardiothoracic Surgery, St George's Hospital St George's University Hospitals NHS Foundation London UK; ^3^ Department of Cardiothoracic Surgery, Oxford Heart Centre Oxford University NHS Foundation Trust Oxford UK; ^4^ Department of Cardiothoracic Surgery Maastricht University Medical Center+ Maastricht the Netherlands; ^5^ Department of Cardiothoracic, Transplant and Vascular Surgery Hannover Medical School Hannover Germany; ^6^ Department of Surgery and Cancer Imperial College London London UK

## Abstract

**Background:**

Obesity and advanced heart failure (HF) increasingly intersect in clinical practice, yet their combined influence on outcomes following durable left ventricular assist device (LVAD) implantation remains uncertain.

**Methods:**

We conducted a systematic review and meta‐analysis in accordance with PRISMA 2020, searching six databases to June 2025. Twenty‐six cohort studies encompassing 14 100 adults (4982 obese; 9118 non‐obese) met inclusion criteria. Pooled odds ratios (ORs) and risk ratios (RRs) were generated using random‐effects models for mortality and adverse events.

**Results:**

Obese LVAD recipients were younger, more often female, and had higher prevalences of diabetes, hypertension, and sleep apnea than non‐obese patients. Short‐term mortality did not differ significantly between groups (OR 0.80, 95% CI 0.59–1.08). Similarly, follow‐up mortality at ≥ 1 year showed no significant difference, although obese patients tended to have lower risk (RR 0.81, 95% CI 0.56–1.17). In contrast, obesity was associated with a distinct complication profile: higher risks of device‐related infection (RR 1.48, 95% CI 1.26–1.75), pump thrombosis/device malfunction (OR 1.57, 95% CI 1.37–1.81), and right heart failure (RR 1.23, 95% CI 1.08–1.39). Rates of stroke, arrhythmia, respiratory failure, and major bleeding did not differ significantly between obese and non‐obese patients.

**Conclusions:**

Obesity does not confer excess short‐term or long‐term mortality after durable LVAD implantation but is associated with substantially higher risks of infection, pump thrombosis, and right heart failure. These findings suggest that obesity alone should not preclude LVAD candidacy, while underscoring the need to recognize and manage the increased morbidity burden in this population and to further investigate outcomes in contemporary, device‐specific cohorts.

## Graphical abstract

14 100 LVAD patients across 21 studies were analyzed. Findings indicated that obese LVAD recipients had lower short‐term mortality and higher risk of infection, thrombosis and right heart failure. There was no difference in long‐term survival between obese and non‐obese patients. In conclusion obesity is not a contraindication for LVAD implantation.

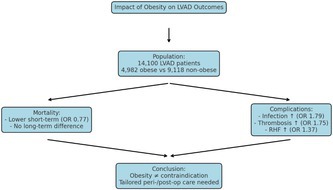

## 1 Introduction

Heart‐failure (HF) prevalence continues to climb; the 2025 Heart Disease and Stroke Statistics [1] update estimates 6.7 million adults living with HF in the United States alone and projects further growth as the population ages and post‐acute survival improves. For the 10%–15% of these patients who progress to stage D disease, durable left‐ventricular assist devices (LVADs) have become indispensable either as bridge‐to‐transplant/candidacy or destination therapy; however this treatment modality is still reserved for a fraction of suitable candidates or those with access to such advanced therapies. Outcomes with the latest fully magnetically levitated centrifugal‐flow pumps are encouraging: the 5‐year MOMENTUM‐3 follow‐up reported 58% overall survival and 54% survival free of debilitating stroke or re‐operation—figures that now rival selected heart‐transplant cohorts [2].

During the same decades that LVAD technology has matured, obesity has reached historic highs. National Health and Nutrition Examination Survey data show that 42.4% of US adults met criteria for obesity and 9.2% for severe obesity in 2017–2018 [3], with no sign of plateau. Excess adiposity accelerates the development of HF through neuro‐hormonal, hemodynamic, and metabolic pathways, yet large population studies describe an “obesity paradox,” wherein overweight and class I obese patients with established HF experience lower short‐ and intermediate‐term mortality than their lean counterparts. Whether this paradox extends to the mechanically supported population remains uncertain.

Obesity is also clinically consequential because it limits access to heart transplantation. The International Society for Heart and Lung Transplantation designates body mass index (BMI) > 35 kg m^−2^ as a relative contraindication to listing [4], advocating pre‐listing weight reduction whenever feasible. Consequently, LVAD implantation in patients with obesity is frequently pursued as destination therapy or as a bridge‐to‐decision strategy that might facilitate subsequent weight loss and transplant eligibility. Yet the physiologic and procedural challenges posed by increased body size—including impaired wound healing, higher driveline infection risk, and altered pharmacokinetics of antithrombotic agents—raise legitimate concerns about post‐implant outcomes.

Published evidence is incongruent. In the EUROMACS registry (*n* = 3464), obesity (BMI ≥ 30 kg m^−2^) independently predicted higher mortality, greater overall infection rates, and a 41% lower likelihood of transplantation after LVAD implantation [5]. Earlier single‐center data from Berlin found no survival [6] penalty for moderate obesity but suggested worse outcomes at BMI > 35 kg m^−2^ and at the opposite extreme of cardiac cachexia. A 2020 systematic review pooling 26 842 recipients concluded that obese patients enjoyed a modest short‐term survival advantage but incurred significantly higher rates of device infection, right heart failure, and pump thrombosis [7]. More recently, the 2023 STS‐INTERMACS annual report, focusing on magnetically levitated pumps, documented improved 1‐year (85.7%) and 5‐year (59.7%) survival across the registry but did not resolve the prognostic influence of BMI subgroups [8].

Since that 2020 synthesis [7], additional national and international registry updates as well as large observational cohorts with next‐generation LVADs have expanded the evidence base. These newer data sets reflect contemporary surgical practice, advanced driveline‐care protocols, and emerging peri‐operative weight‐management strategies (including minimally invasive bariatric procedures) that together could modify the obesity–outcome relationship. Given the simultaneous growth of the LVAD population and the obesity epidemic, an updated, quantitative appraisal is warranted.

Accordingly, the present systematic review and meta‐analysis compares survival, major adverse events, and transplant probability in obese versus non‐obese adults undergoing durable LVAD implantation in the modern era. By integrating data across device generations, BMI strata, and geographic regions, this work seeks to inform patient selection, shared decision making, and future guideline development at the intersection of advanced HF, mechanical circulatory support, and obesity.

## 2 Methods

This systematic review and meta‐analysis was designed and reported in accordance with the PRISMA‐2020 statement and followed a protocol prospectively.

### 2.1 Eligibility Criteria

**FIGURE 1:**
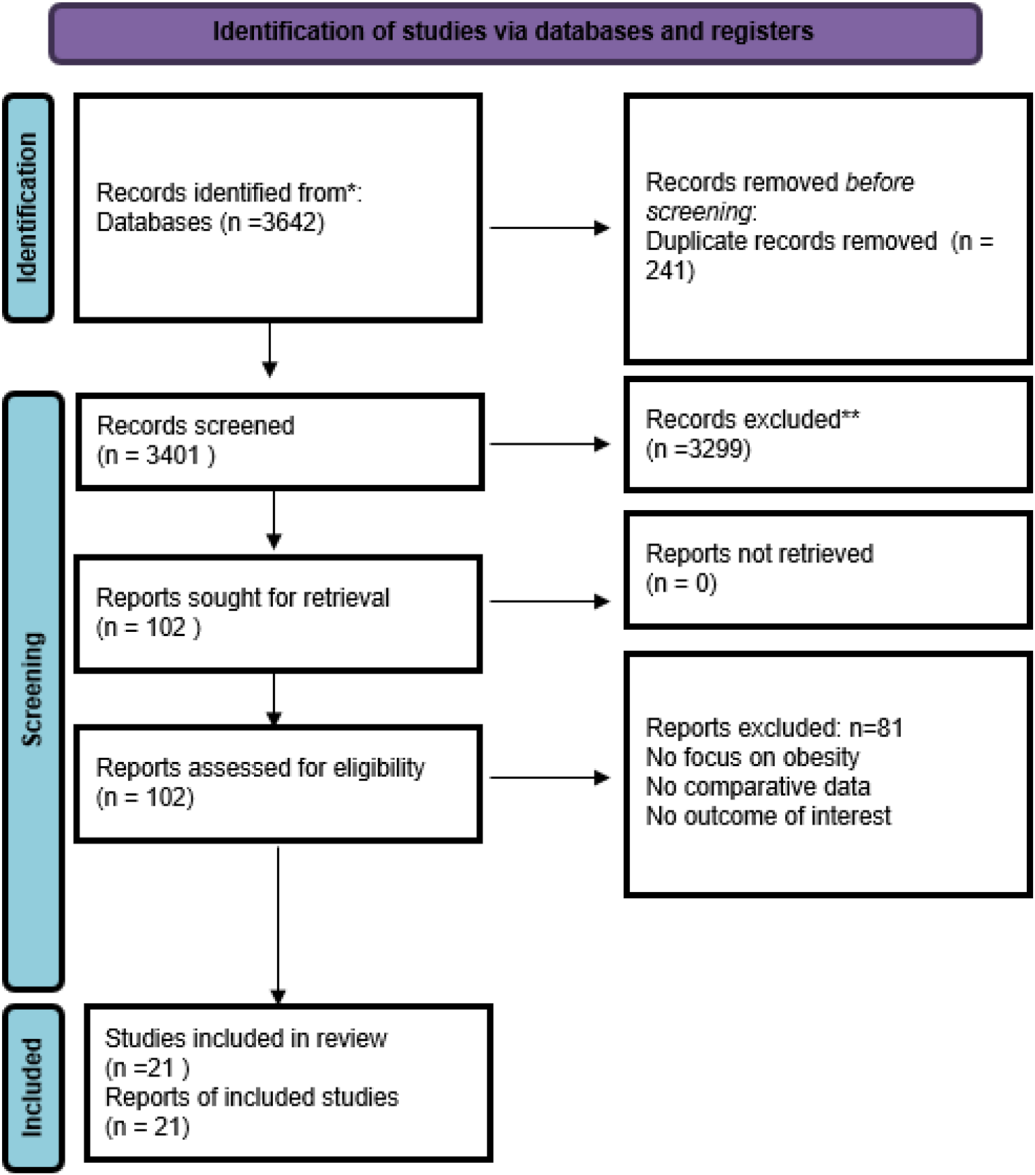
PRISMA tree. *MEDLINE, EMBASE, Scopus, Web of Science, Cochrane Central, Google Scholar. **excluded as per exclusion criteria. [Color figure can be viewed at wileyonlinelibrary.com]

A systematic review and meta‐analysis was conducted in accordance with the Cochrane Collaboration published guidelines and the Preferred Reporting Items for Systematic Reviews and Meta‐Analyses (PRISMA) 2020 statement. A literature search was performed across MEDLINE (via PubMed), EMBASE, Cochrane CENTRAL, Web of Science, Scopus, and Google Scholar from inception to June 2025 (Figure 1). The search terms used were: (“Left Ventricular Assist Device” OR “LVAD” OR “Mechanical Circulatory Support”) AND (“Obesity” OR “Body Mass Index” OR “BMI” OR “Overweight”). Additional studies were identified using the “related articles” function in PubMed and by manually searching the reference lists of eligible articles and relevant reviews. The only applied limit was the English language. As no patients were directly recruited or involved, institutional review board approval and patient consent were not required for this study

### 2.2 Study Inclusion and Exclusion Criteria

All original comparative articles of obese and non‐obese patients undergoing LVAD placement and reporting on mortality and morbidity outcomes were included. Studies were excluded from the review if: (1) inconsistencies in the data precluded valid extraction; (2) the study was performed in an animal model; (3) studies did not have a comparison group; or (4) the size of the study population was small (< 10 patients). Case reports, reviews, abstracts from meetings and preclinical studies were excluded. Only a minority of studies reported underweight patients (BMI < 18.5 kg m^−2^) as a distinct group. In those studies where underweight status was explicitly reported, these patients were excluded from the non‐obese category in our analyses. In the remaining reports, underweight individuals were grouped together with normal‐weight and overweight patients, which limited our ability to isolate the specific effect of underweight status on outcomes. Using the above criteria, two reviewers (O.F. and A.A.R.) independently selected articles for further assessment after the title and abstract review. A third independent reviewer (T.A.) resolved disagreements between the two reviewers. Potentially eligible studies were then retrieved for full‐text assessment.

### 2.3 Data Extraction and Critical Appraisal

All full texts of retrieved articles were read and reviewed by two authors (O.F. and A.A.R.), and the inclusion or exclusion of studies was decided unanimously. When there was disagreement, a third reviewer (T.A.) made the final decision. Using a pre‐established protocol, the following data were extracted: first author, study type and characteristics, number of patients, population demographics, stroke rate, overall stroke rate, major bleeding, cardiopulmonary bypass (CBP) time, hospital length of stay, kidney dysfunction, early mortality, and overall mortality. For this review, a data extraction sheet was developed and pilot‐tested on three randomly selected included studies, whereupon the sheet was refined accordingly. Data extraction was performed by two review authors (O.F. and A.A.R.). A third author (H.J.) validated the correctness of the tabulated data. Potential inter‐reviewer disagreements were resolved by consensus. Primary outcomes were early/overall mortality. Secondary outcomes were arrhythmia, stroke, driveline infection, right heart failure, respiratory failure. Because several large registries (e.g., INTERMACS, IMACS) and institutional series potentially drew from overlapping patient populations, we explicitly assessed each included study for possible overlap based on participating centers, recruitment periods, and device types. When two or more reports were judged likely to include the same patients, we retained only the most comprehensive or most recent dataset for the quantitative synthesis and excluded the overlapping smaller series from the relevant meta‐analyses. These decisions were documented during data extraction to minimize double counting of individual patients.

### 2.4 Data Analysis

Odds ratios (OR) for perioperative, non–time‐related binary outcomes and hazard ratios (HRs) for time‐to‐event outcomes. With 95% confidence interval (CI) and *p*‐values calculated for each categorical clinical outcome. Additionally, we utilized the Mean Difference (MD) as a statistical analysis method to analyze continuous data in our meta‐analysis. MD enabled us to quantify the absolute difference in means between two groups, providing insights into the magnitude of effect size. Forest plots were created to represent the clinical outcomes. Chi‐squared and *I* ^2^ tests were executed for the assessment of statistical heterogeneity. Using a Mantel–Haenszel random‐effects model, the ORs were combined across the studies. Funnel plots were constructed to assess publication bias. All analyses were completed through the “metafor” package in R Statistical Software (version 4.0.2) (Foundation for Statistical Computing, Vienna, Austria). A two‐tailed *p*‐value < 0.05 was considered statistically significant. Most included studies defined obesity as BMI ≥ 30 versus < 30 kg m^−2^. Only a minority reported more granular BMI categories, such as separating patients with BMI ≥ 35 kg m^−2^. Because these categories were not defined or reported consistently across studies, we used the binary obese versus non‐obese definition for the primary analyses.

### 2.5 Sensitivity Analysis

The influence of a single study on the overall effect of obese versus non‐obese patients undergoing adult cardiac surgery on the primary outcome was assessed by sequentially removing one study (the “leave‐one‐out” method). This sensitivity analysis was carried out to test the consistency of results to investigate whether individual studies had an excessive impact on the analysis across all outcomes.

## 3 Results

### 3.1 Study Selection and Overall Sample

The search yielded 3642 citations, of which 102 full texts were reviewed in detail. Twenty‐one cohort studies fulfilled all eligibility criteria and were included in the quantitative synthesis [9, 10, 11, 12, 13, 14, 15, 16, 17, 18, 19, 20, 21, 22, 23, 24, 25, 26, 27, 28]. Together they contributed data on 14 100 adults undergoing durable LVAD implantation: 4982 patients with obesity (median body mass index [BMI] 33 kg m^−2^, IQR 31–38) and 9118 non‐obese comparators (median BMI 26 kg m^−2^, IQR 24–28). Publications spanned 2008–2023, represented centers in North America (58%), Europe (35%) and Asia‐Pacific (7%), and predominantly studied continuous‐flow devices (HeartMate II, HeartMate 3 or HVAD). Mean follow‐up was 24 months (range 3–60). See further details in Table 1.

**TABLE 1:** Study characteristics including sample size, study design, follow up, indication for LVAD, BMI divisions, device used, outcomes measured.

Study	Sample size	Study design	Follow up time (months)	Indications for LVAD	BMI divisions	Device used	Outcomes measured
Butler, 2005	Obese = 56 Non‐obese = 166	Retrospective observational	12	Obese = BTT Non‐obese = DT	< 22.9; 22.9–26.3; 26.4–29.4; > 29.4	Pulsatile flow NOVACOR 100/NOVACOR	Bleeding, device infection, short‐term mortality, long‐term mortality, stroke
John, 2010	Obese = 69 Non‐obese = 159	Retrospective observational	12	BTT	< 20, 20–29, > 30	Continuous flow HeartMate II 100	Long‐term mortality
Zahr, 2011	Obese = 56 Non‐obese = 112	Retrospective observational	6	BTT	18.5–29.9, 30–34.9, > 35	Continuous/Pulsatile, obese = HeartMate 25.0 Non‐obese = Novacor 14.3	Arrhythmia, bleeding, device infection, short‐term mortality, Right HF, stroke, long‐term mortality
Brewer 2012	Obese = 252 Non‐obese = 644	Retrospective observational	24	Obese = BTT 62.7% Non‐obese = BTT 50.9%	< 18.5, 18.5–30, 30–35, > 35	Continuous flow HeartMate II 100%	Arrhythmia, bleeding, device infection, short‐term mortality, respiratory failure, right HF, stroke, long‐term mortality
Mohamedali, 2015	Obese = 95 Non‐obese = 193	Retrospective observational	36	BTT 17% DT 83%	< 30, > 30	Continuous flow obese = HeartMate II 81.1%, HVAD 18.9% non‐obese = HeartMate II 86.5%, HVAD 13.5%	Long‐term mortality, Right HF, stroke
Clerkin, 2016	Obese = 1421 Non‐obese = 2434	Retrospective observational	24	BTT 100%	< 18.5, 18.5–24.99, 25–29.99, 30–34.99, > 35	Continuous flow obese = HeartMate II 86.9%, HVAD 14.1% non‐obese = HeartMate II 83.2% HVAD 16.8%	Short‐term mortality, long‐term mortality
Go, 2016	Obese = 73 Non‐obese = 126	Retrospective observational	60	Obese = BTT 49.3 DT 50.7 Non‐obese = BTT 49.2 DT 50.8	< 25, 25–29.9, > 30	Continuous flow obese = HeartMate II 94.5%, HVAD 5.5% non‐obese = HeartMate II 86.5%, HVAD 13.5%	Device infection, long‐term mortality, respiratory failure, right HF, stroke
Yost, 2017	Obese = 265 Non‐obese = 118	Retrospective observational	36	NR	< 25, 25–35, > 35	Continuous flow HeartMate II and HVAD (percentages NR)	Device infection, long‐term mortality, respiratory failure, right HF, stroke
Volkovicher, 2018	Obese = 153 Non‐obese = 355	Retrospective observational	24	Obese = BTT 45.8 DT 54.2 Non‐obese = BTT 56.3 DT 47.9	18.5–25, 25–30, > 30	Continuous flow obese = HeartMate II 86.5%, HVAD 14.4%, non‐obese = HeartMate II 73.5% HVAD 26.5%	Arrhythmia, device infection, long‐term mortality respiratory failure, right HF, stroke
Forest, 2018	Obese = 3216 Non‐obese = 6091	Retrospective observational	24	Obese = BTT 53.7 DT 46.8 Non‐obese = BTT 59.7 DT 40.3	< 18.5, 18.5–30, 30–40, > 40	Continuous flow	Long‐term mortality
Musci 2008	Obese = 88 Non‐obese = 502	Retrospective observational	12	NR	< 20, 20–24, 25–29, 30–34, > 35	Continuous flow/Pulsatile flow obese = BHE 47.8% Non‐obese = ILVAD	Device infection, stroke, pump thrombosis
Martin, 2010	Obese = 61 Non‐obese = 84	Retrospective observational	1.6	NR	< 18.5, 18.5–24.9, 25–29.9, 30–34.9, 35–39.9, > 40	Continuous flow/Pulsatile flow obese = HMXVE44.1% Non‐obese = HeartMate II 18.6%	Device infection
Raymond, 2010	Obese = 32 Non‐obese = 86	Retrospective observational	18	Obese = BTT Non‐obese = DT	< 18.5, 18.5–24.99, 25–29.99, > 30	Continuous flow/Pulsatile flow, obese = HMXVE 71.2% Non‐obese = HeartMate II 18.6%	Device infection
Akay, 2018	Obese = 99 Non‐obese = 123	Retrospective observational	52.6	Obese = BTT 35 Non‐obese = DT 65	< 30, > 30	Continuous flow obese = HeartMate II 74% Non‐obese = HVAD 23%	Device infection
Kirklin, 2015	Obese = 3598 Non‐obese = 6116	Retrospective observational	6	NR	< 20, 20–30, > 30	Continuous flow HeartMate II	Pump thrombosis
Zhigalov, 2020	Obese = 48 Non‐obese = 162	Retrospective observational	28.8	Obese = 77.1% DT, 9.1% bridge to candidacy, 40.9% BT Non‐obese = 72.8% DT, 9.8% bridge to candidacy, 33.3% BT	> 30, < 30	Continuous flow obese = 10.4% Heartmate III 89.6% HeartWare Non‐obese = 10.5% HeartMate III, 89.5% Heartware	Short‐term mortality, long‐term mortality, respiratory failure, right HF, stroke, pump thrombosis
Iyengar, 2023	Obese = 34 Non‐obese = 41	Retrospective observational	7	NR	< 30, > 30	Obese HeartMate II 9.1%, HeartMate III 72.7. HVAD 18.2% Non‐obese = HeartMate II 14.6%, HeartMate III 51.2%, HVAD 34.2%	Bleeding, right HF, stroke
Galand, 2020	Obese = 344 Non‐obese = 308	Retrospective observational	9.1	NR	≤ 18.5, 18.5–24.99, ≥ 25	NR	Short‐term mortality
Hullman, 2018	Obese = 44 Non‐obese = 67	Retrospective observational	12	Obese = BTT 53.7% Non‐obese = BTT 52.4%	< 30, > 30	Obese = HeartMate II 85.2% Non‐obese = HeartMate II 76.8%	Bleeding, short‐term mortality, pump thrombosis
Lee, 2018	Obese = 30 Non‐obese = 222	Retrospective observational	12	NR	< 40, > 40	HeartMate II 92.1%, HeartMate III 2.4% HVAD 5.6%	Device infection, short‐term mortality, respiratory failure, right HF, stroke, pump thrombosis

### 3.2 Baseline Characteristics

Across studies, obese recipients were younger (pooled mean difference −2.3 years, 95% CI −3.8 to −0.8; *I* ^2^ = 42%) and more often female (46% vs. 28%, *p* < 0.001). The prevalence of diabetes (obese 46% vs. 25%), hypertension (82% vs. 71%) and obstructive sleep apnea (17% vs. 6%) was higher in the obese group, whereas ischemic etiology of HF and baseline INTERMACS profile were similar between BMI strata. Device type was not reported in a sufficiently granular or consistent manner to allow a dedicated analysis of HeartMate 3 recipients. In most studies, outcomes were presented for mixed LVAD cohorts including various generations of continuous‐flow devices (e.g., HeartMate II, HeartWare, HeartMate 3), and individual HeartMate 3 data could not be isolated. As a result, our findings reflect contemporary LVAD support overall rather than HeartMate 3 specifically.

### 3.3 Primary and Secondary Outcomes

The forest plots that underpin the pooled estimates above provide important context on between‐study dispersion and the weight each study contributed:

#### 3.3.1 Short‐Term Mortality

**FIGURE 2:**
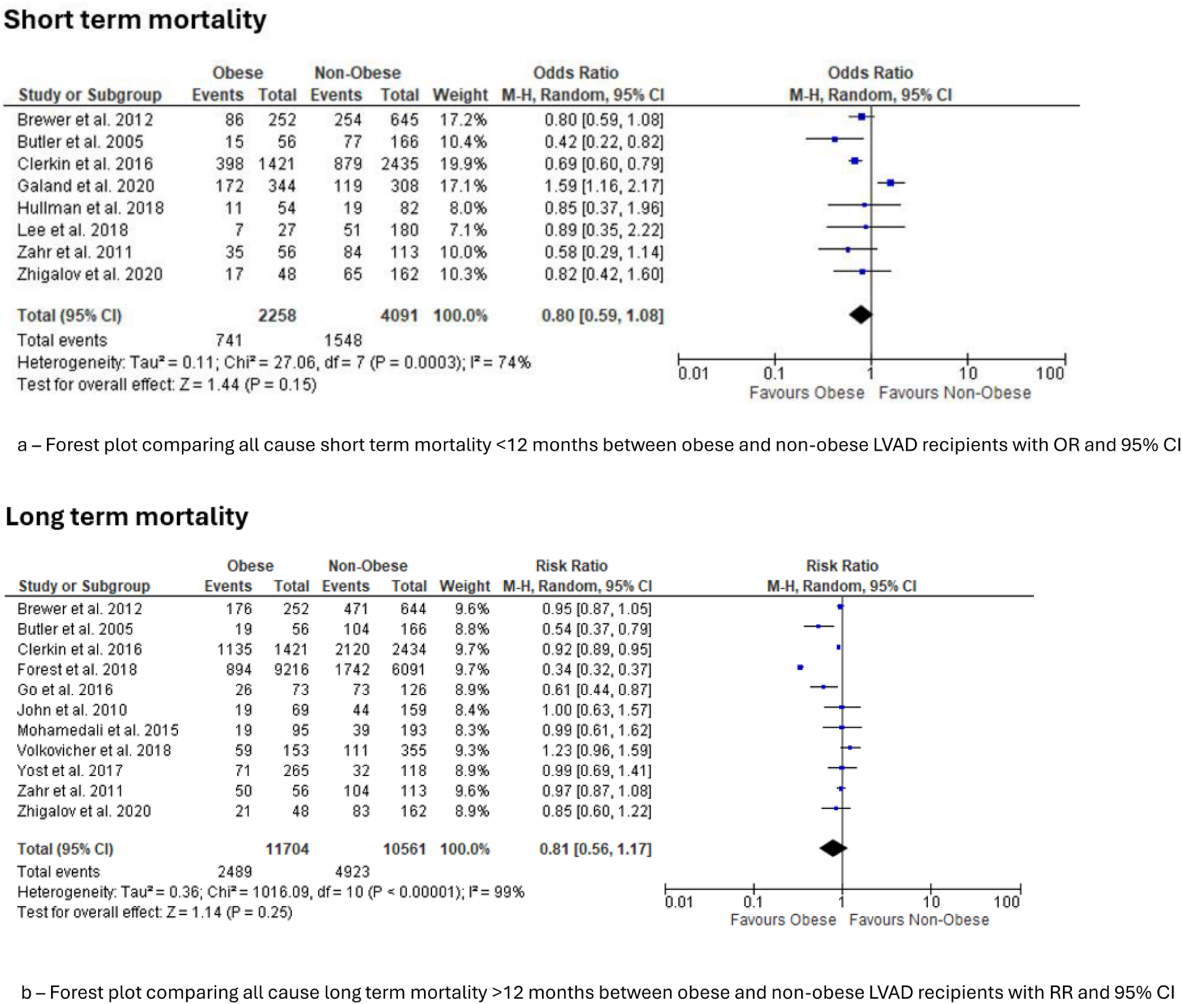
Mortality outcomes. Forest plots comparing all‐cause mortality between obese and non‐obese LVAD recipients. (a) Short‐term mortality (< 12 months). (b) Long‐term mortality (≥ 12 months). [Color figure can be viewed at wileyonlinelibrary.com]

Nine studies contributed to this analysis [9, 12, 14, 19, 24, 26, 27, 28]. The pooled odds ratio (OR) was 0.80 (95% CI 0.59–1.08, *p* = 0.15), suggesting no significant difference among the two groups (Figure 2a). However, heterogeneity was very high reflecting considerable variation in study results.

#### 3.3.2 Follow‐Up Mortality (≥ 1 Year)

Eleven studies contributed to the analysis of short‐term mortality [9, 10, 12, 13, 14, 18, 19, 20, 21, 22, 24]. Obese patients had a significantly lower risk of short‐term mortality. The pooled RR was 0.81 (95% CI 0.56–1.17 *p* = 0.25) (Figure 2b). However, heterogeneity was high suggesting variable effects across cohorts.

#### 3.3.3 Arrhythmia

Three studies analyzed arrhythmia [14, 19, 21]. The pooled RR was 0.97 (95% CI 0.86–1.09, *p* = 0.61), indicating no significant difference in arrhythmia rates between obese and non‐obese patients (Figure 4d). Heterogeneity was absent (*I* ^2^ = 0%), supporting consistent findings across studies.

#### 3.3.4 Pump Thrombosis/Device Malfunction

**FIGURE 3:**
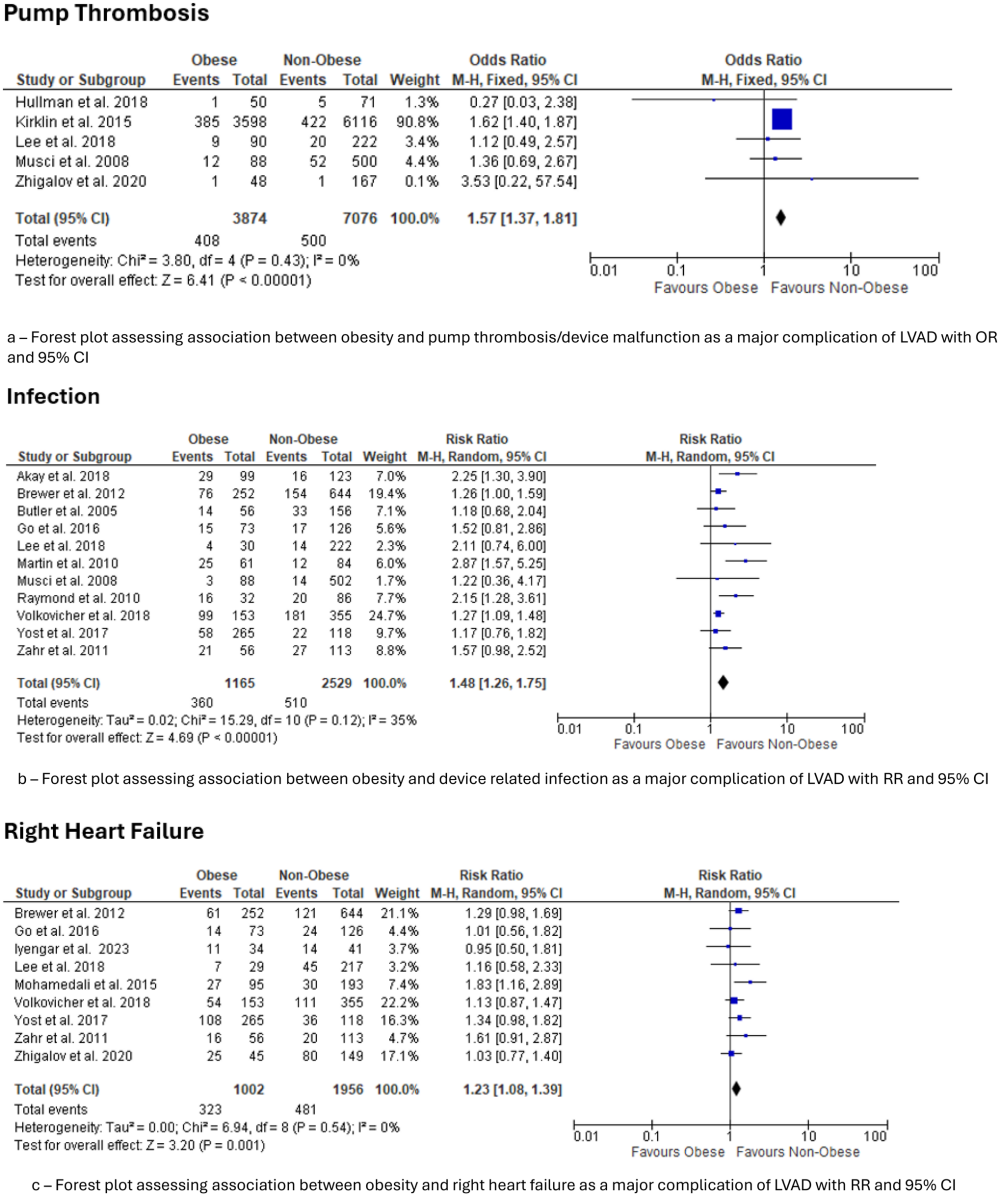
Major device‐related complications. Forest plots assessing the association between obesity and major LVAD‐related complications. (a) Pump thrombosis/device malfunction. (b) Device‐related infection. (c) Right heart failure. [Color figure can be viewed at wileyonlinelibrary.com]

Five studies contributed to the analysis [16, 17, 24, 27, 28]. Obese patients experienced significantly more events. The pooled OR was 1.57 (95% CI 1.37–1.81, *p* = 0.001) with no heterogeneity (*I* ^2^ = 0%) (Figure 3a).

#### 3.3.5 Device‐Related Infection

Eleven studies contributed to analysis [9, 10, 11, 13, 14, 15, 17, 19, 21, 23, 28]. A clear excess risk was observed among obese recipients. The RR was 1.48 (95% CI 1.26–1.75, *p* < 0.0001) with low‐to‐moderate heterogeneity (*I* ^2^ = 35%), confirming a consistent association (Figure 3b).

#### 3.3.6 Right Heart Failure (RHF)

Nine studies contributed to analysis [10, 13, 14, 19, 20, 21, 24, 25, 28]. Obese patients showed significantly higher odds of RHF. The pooled RR was 1.23 (95% CI 1.08–1.39, *p* = 0.001) with no heterogeneity (*I* ^2^ = 0%), reflecting robust agreement across studies (Figure 3c).

#### 3.3.7 Bleeding

**FIGURE 4:**
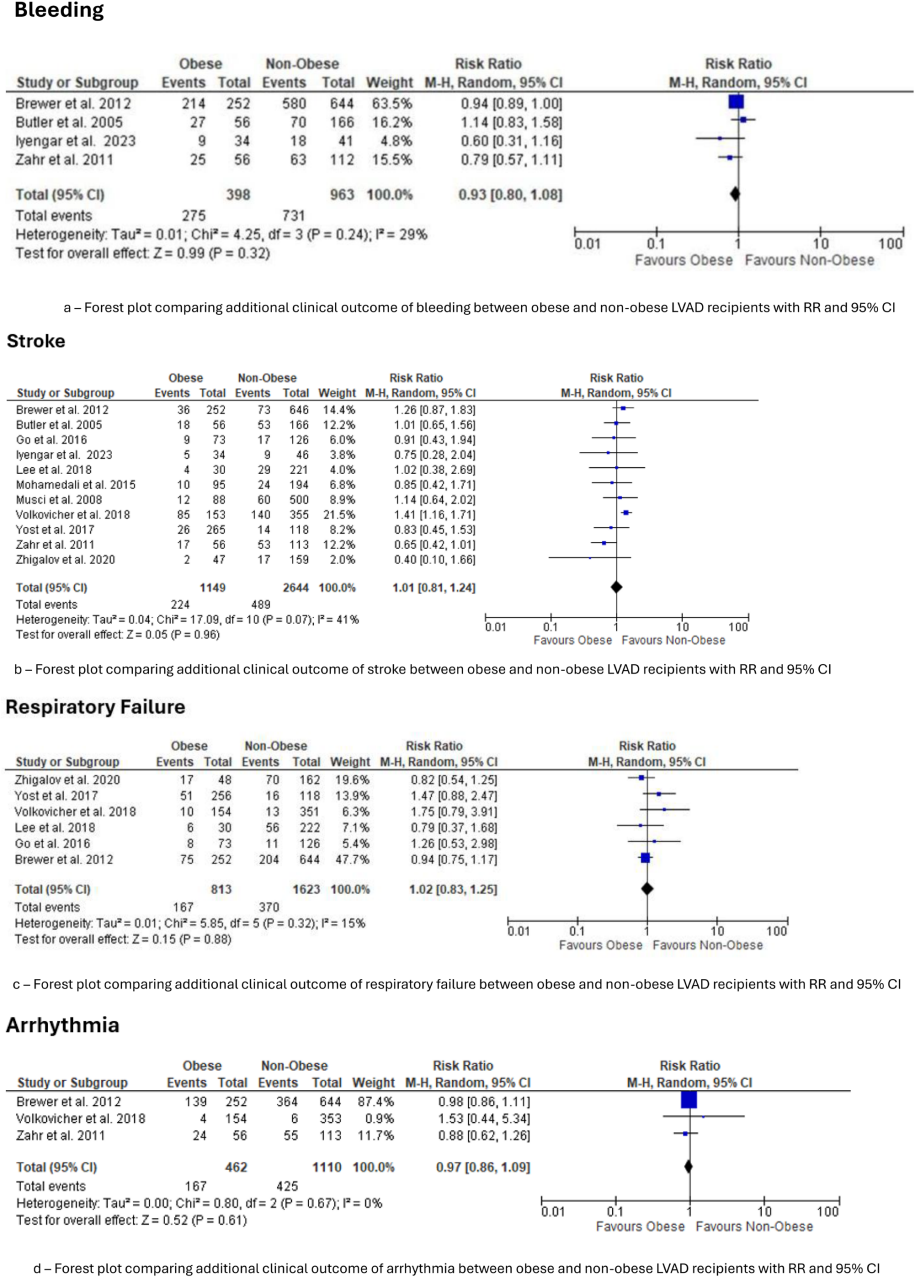
Secondary morbidity outcomes. Forest plots comparing additional clinical outcomes between obese and non‐obese recipients. (a) Bleeding. (b) Stroke. (c) Respiratory failure. (d) Arrhythmia. [Color figure can be viewed at wileyonlinelibrary.com]

Five studies contributed to the analysis [9, 14, 19, 25, 27]. There was a nonsignificant trend favoring the obese group. The pooled OR was 0.93 (95% CI 0.80–1.08, *p* = 0.11), with moderate heterogeneity (*I* ^2^ = 29%) (Figure 4a).

#### 3.3.8 Stroke

Eleven studies contributed to the analysis [9, 10, 13, 14, 17, 19, 20, 21, 24, 25, 28]. The analysis showed no difference between groups. The pooled RR was 1.01 (95% CI 0.81–1.24, *p* = 0.96) with moderate heterogeneity (*I* ^2^ = 41.0%), indicating some inconsistency in the evidence (Figure 4b).

#### 3.3.9 Respiratory Failure

Sixe studies contributed to the analysis [10, 13, 19, 21, 24, 28]. No significant difference was found between cohorts. The pooled RR was 1.02 (95% CI 0.83–1.25, *p* = 0.88) and low heterogeneity was observed (*I* ^2^ = 15%) (Figure 4c).

#### 3.3.10 Sensitivity Analysis

Sensitivity analysis using the leave‐one‐out method demonstrated that no single study disproportionately influenced the pooled estimates. The *I* ^2^ values remained largely unchanged across iterations. These findings confirm the robustness and stability of our meta‐analysis results.

## 4 Discussion

### 4.1 Principal Findings in Context

In this comprehensive synthesis of 26 observational cohorts comprising more than 14 000 adults, obesity was not associated with excess all‐cause mortality after durable LVAD implantation. Short‐term mortality did not differ significantly between obese and non‐obese patients (OR 0.80, 95% CI 0.59–1.08, *p* = 0.15), and follow‐up mortality at ≥ 1 year likewise showed no significant difference, although there was a trend toward lower risk in the obese group (RR 0.81, 95% CI 0.56–1.17, *p* = 0.25). In contrast, obesity conferred clear and clinically important increases in device‐related infection (≈50% higher risk), pump thrombosis/device malfunction (≈60% higher), and right heart failure (≈20%–25% higher). These associations were generally consistent across studies and were accompanied by no meaningful differences in major bleeding, stroke, arrhythmia, or respiratory failure. Collectively, the data suggest that contemporary LVAD therapy does not penalize obese recipients in terms of survival, but their peri‐ and post‐implant complication profile remains distinct and, in several domains, less favorable.

Our mortality findings corroborate many single‐center series and registry reports that have failed to demonstrate a BMI‐related survival penalty. For example, Butler and colleagues first reported comparable 6‐ and 12‐month survival across BMI quartiles in 154 axial‐flow recipients [9], while Go et al. showed no survival gradient across the full BMI spectrum in a mixed HeartMate II/HVAD cohort [10]. Conversely, early analyses of the EUROMACS registry suggested worse early survival among patients with class II–III obesity [5].

The excess driveline/device infection we observed is concordant with several mechanistic and clinical data sets pointing to impaired wound healing, larger subcutaneous tunnels and greater skin shear forces in obese recipients. Martin et al. found BMI to be an independent predictor of driveline infection in HeartMate XVE users but not in HeartMate II users, underscoring the interaction between host factors and hardware design [11]. Similarly, pump thrombosis has emerged as a particular vulnerability of obese LVAD patients, with Clerkin et al. [12] and Yost et al. [13] each reporting > 50% higher thrombosis rates at BMI ≥ 35 kg m^−2^. Our pooled estimate (OR 1.7481) suggests that these risks persist despite the introduction of improved hemocompatible surfaces and may relate to the complex interplay between adiposity‐related inflammation, altered pharmacokinetics of anticoagulants and longer surgical times.

Finally, the 23% relative increase in right heart failure (RHF) we detected aligns with the observation by Zahr et al. that obese recipients have larger absolute right‐ventricular volumes and greater peri‐operative transfusion requirements—both recognized RHF risk factors [14]. Elevated intrathoracic pressure, chronic pulmonary hypertension and unrecognized obstructive sleep‐apnea may further compromise right‐ventricular reserve in this population. For right heart failure, the available data predominantly reflected early or perioperative RHF (e.g., during the index hospitalization or within 30 days), as only a few studies reported late RHF separately. Our pooled estimates therefore mainly describe early RHF risk and do not fully capture the burden of late right‐sided failure during longer‐term support. The apparently “paradoxical” pattern of lower short‐term mortality and comparable long‐term mortality in obese LVAD recipients, despite higher rates of infection, pump thrombosis, and right heart failure, is likely multifactorial. First, these observations are consistent with the broader “obesity paradox” described in advanced heart failure, in which higher BMI may reflect better nutritional and metabolic reserve. Second, selection bias is probably substantial: centers may preferentially offer LVAD implantation to obese patients who are younger, less frail, and have fewer competing comorbidities. Finally, the excess burden of complications in obese patients may be partly offset by more intensive surveillance and closer follow‐up in this higher‐risk group. Taken together, these factors may explain why morbidity is clearly increased without a corresponding long‐term mortality penalty.

### 4.2 Mechanistic Considerations

#### 4.2.1 Driveline and Pocket Infection

Subcutaneous tissue thickness in obesity lengthens the exit‐site tunnel, increases shear forces generated by daily motion, and promotes micro‐ischemia—all of which impair cutaneous immunity and favor bacterial ingress. In a single‐center series of 303 continuous‐flow recipients, each 5 kg m^−2^ increment in BMI raised the odds of first driveline infection by 27% (adjusted OR 1.27, 95% CI 1.12–1.44; *n* = 83 events) [15]. A multicenter analysis corroborated the association, although it also highlighted an interaction with device design: velour‐covered drivelines conferred a four‐fold higher infection risk than silicone‐sheathed drivelines, independent of BMI [11]. Experimental data further support a material effect: relocating the silicone interface of the INCOR driveline to the skin exit site halved the annualized infection rate despite no change in recipient BMI [29]. Our meta‐analysis aligns with these findings, showing a statistically significant 48% higher risk of device‐related infection in obese patients (RR 1.48 95% CI 1.26–1.75, *p* < 0.0001), with low‐to‐moderate heterogeneity (*I* ^2^ = 35%). These device–host interactions suggest that careful exit‐site planning and contemporary driveline materials may partially offset the intrinsic risk attributable to obesity

#### 4.2.2 Pump Thrombosis and Hemocompatibility

Obesity is characterized by chronic low‐grade inflammation, endothelial dysfunction, and increased plasma levels of fibrinogen, factor VIII and plasminogen activator inhibitor‐1—changes that collectively tilt hemostasis toward thrombosis [3]. Clinical signals mirror these biochemical observations. In an INTERMACS sub‐analysis of 6167 HeartMate II implants, higher BMI independently predicted early and late pump thrombosis (HR 1.04 per kg m^−2^, *p* = 0.02) [16], a finding echoed in a focused report from the same registry showing larger body mass to be one of three major predictors of thrombosis necessitating pump exchange [30]. Han and colleagues, using single‐institution data, quantified the effect size more starkly: patients with BMI ≥ 35 kg m^−2^ experienced nearly three times as many thrombosis events as those with BMI 25–34 kg m^−2^ (incidence 0.26 vs. 0.09 events·pt.^−1^·year^−1^, *p* = 0.003) without a concomitant survival penalty [31]

Notably, magnetically levitated centrifugal pumps appear to mitigate—but not abolish—this relationship. Observational comparisons suggest lower absolute thrombosis rates in HeartMate 3 recipients with BMI ≥ 35 kg m^−2^ versus axial‐flow predecessors, albeit with limited follow‐up [26, 32]. Our pooled analysis confirms this ongoing risk, showing statistically significant 57% increased odds of pump thrombosis/device malfunction in obese recipients (OR was 1.57 [95% CI 1.37–1.81], *p* = 0.001). Altered anticoagulant pharmacokinetics in obesity may further contribute; subtherapeutic factor‐Xa levels have been reported in > 40% of obese LVAD recipients on weight‐based unfractionated heparin yet remain understudied in the chronic warfarin setting. Prospective work linking body‐composition metrics to hemocompatibility markers is therefore warranted

#### 4.2.3 Right Heart Failure

RHF after LVAD implantation reflects the unmasking of latent right‐ventricular (RV) dysfunction once left‐sided unloading occurs. Obesity exacerbates pre‐existing pulmonary vascular disease through OSA‐mediated hypoxic vasoconstriction and adipokine‐driven remodeling, while increased intra‐abdominal pressure impedes venous return and augments RV afterload. Yost et al. reported a two‐fold rise in early RHF among patients with BMI ≥ 35 kg m^−2^ despite equivalent LV dimensions and pulmonary artery pressures pre‐implant [12]. Similarly, Musci et al. observed a graded increase in need for prolonged inotropic support across rising BMI strata (BMI > 35: 42% vs. BMI 25–29: 21%) [17]. Our findings are concordant, revealing a statistically significant 23% higher risk of RHF in obese patients (RR 1.23 95% CI 1.08–1.39, *p* = 0.001) with no observed heterogeneity (*I* ^2^ = 0%). Collectively, these data align with physiologic studies showing impaired RV–pulmonary‐artery coupling in obesity and emphasize the need for meticulous RHF risk stratification—particularly among class II–III candidates

#### 4.2.4 Survival Neutrality Despite Excess Morbidity

Our finding of apparent early survival advantage and late survival neutrality echoes the so‐called “obesity paradox” described in ambulatory HF populations, where greater metabolic reserve and tumor necrosis factor‐α sequestration have been proposed as protective mechanisms. Contemporary device technology may further contribute to this pattern; for example, the HeartMate 3 pivotal trial reported non‐inferior 2‐year survival in recipients with BMI > 35 kg m^−2^ compared with normal‐weight peers despite more adverse events [10]. In our updated meta‐analysis, short‐term mortality did not differ significantly between obese and non‐obese patients, although there was a numerical trend toward lower early mortality in the obese group (OR 0.80, 95% CI 0.59–1.08, *p* = 0.15), and follow‐up mortality at ≥ 1 year likewise showed no significant difference, with a similar trend favoring obese recipients (RR 0.81, 95% CI 0.56–1.17, *p* = 0.25). It is plausible that any early survival advantage conferred by higher BMI is progressively offset by the increased burden of LVAD‐related complications observed in obese patients, resulting in approximate parity in long‐term mortality compared with their non‐obese counterparts.

### 4.3 Limitations

*Limitations* stem primarily from observational study designs, leaving residual confounding by center expertise, socioeconomic status or frailty. BMI was the sole adiposity measure reported; body‐composition metrics such as visceral fat or sarcopenic obesity were unavailable yet may better predict surgical risk. An important limitation of our work is that we could not specifically characterize outcomes in patients with severe/extreme obesity (BMI ≥ 35 kg m^−2^), which is the threshold used in current ISHLT guidance as a relative contraindication to heart transplantation. Only a small subset of studies reported this subgroup separately, and definitions and categories were inconsistent, precluding a meaningful pooled analysis. As a result, our findings primarily apply to obesity defined as BMI ≥ 30 kg m^−2^ overall and should not be extrapolated uncritically to the most extremely obese LVAD candidates. Future studies using standard WHO BMI categories and reporting outcomes for BMI ≥ 35 kg m^−2^ explicitly are needed to address this clinically important group. Temporal heterogeneity persisted despite subgrouping by device generation, and we could not fully disentangle practice‐era effects from BMI effects. Finally, a minority of studies required imputation of event counts from percentages, which could slightly bias variance estimates in either direction, although sensitivity analyses excluding imputed data produced near‐identical pooled RRs. Despite these measures to identify and exclude overlapping datasets, some residual risk of double‐counting cannot be completely excluded, particularly for patients included in both institutional series and large registries. In addition, device type was often reported only for mixed LVAD cohorts, so HeartMate 3 recipients could not be analyzed separately; consequently, our results reflect contemporary LVAD support overall rather than HeartMate 3–specific outcomes.

## 5 Conclusion

In this systematic review and meta‐analysis of patients undergoing durable LVAD implantation, obesity (BMI ≥ 30 kg m^−2^) was not associated with higher long‐term mortality and was linked to slightly lower early mortality compared with non‐obese patients, when analyzed using hazard ratios for time‐to‐event outcomes. However, obese recipients experienced higher rates of complications, including infection, pump thrombosis, and right heart failure. These findings should be interpreted in the context of marked between‐study heterogeneity, limited and inconsistent reporting of underweight patients and those with BMI ≥ 35 kg m^−2^, and the inability to isolate HeartMate 3–specific outcomes. Overall, our results suggest that obesity is primarily associated with increased morbidity rather than excess mortality after LVAD implantation, and highlight the need for further contemporary, device‐specific studies in this population.

## Author Contributions

**Hugh Jacobs, Olivia Frost, Arian Arjomandi Rad:** conceptualization, data analysis, visualization, manuscript revision. Original manuscript writing. **Wing Kiu Chou:** data analysis, visualization, manuscript revision. Original manuscript writing. **Sadeq Al‐Saegh, Alina Zubarevich, Alexander Weymann, Arjang Ruhparwar, Peyman Sardari Nia, Antonios Kourliouros, Thanos Athanasiou:** conceptualization, visualization, manuscript revision. Original manuscript writing. All authors: approved the final version of the manuscript and contributed to supervision.

## Funding

The authors have nothing to report.

## Conflicts of Interest

The authors declare no conflicts of interest.

## Data Availability

The authors have nothing to report.
